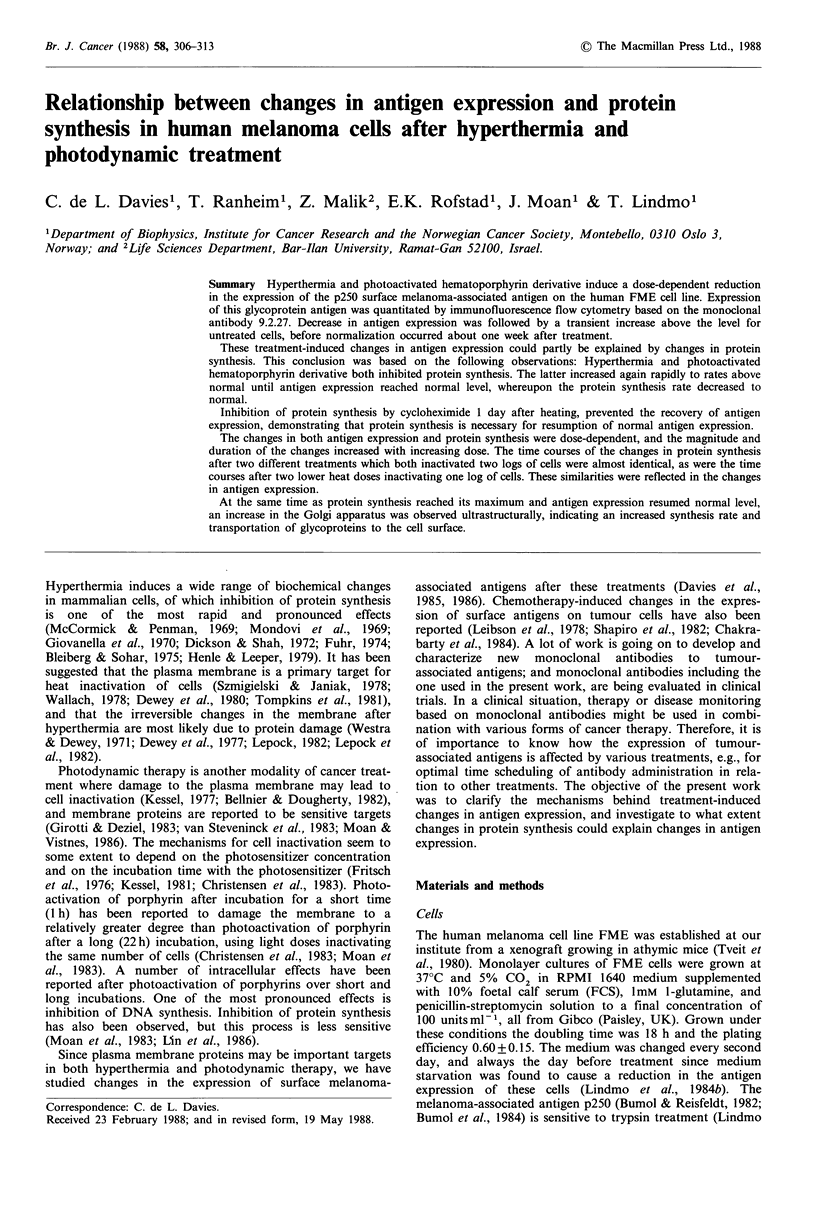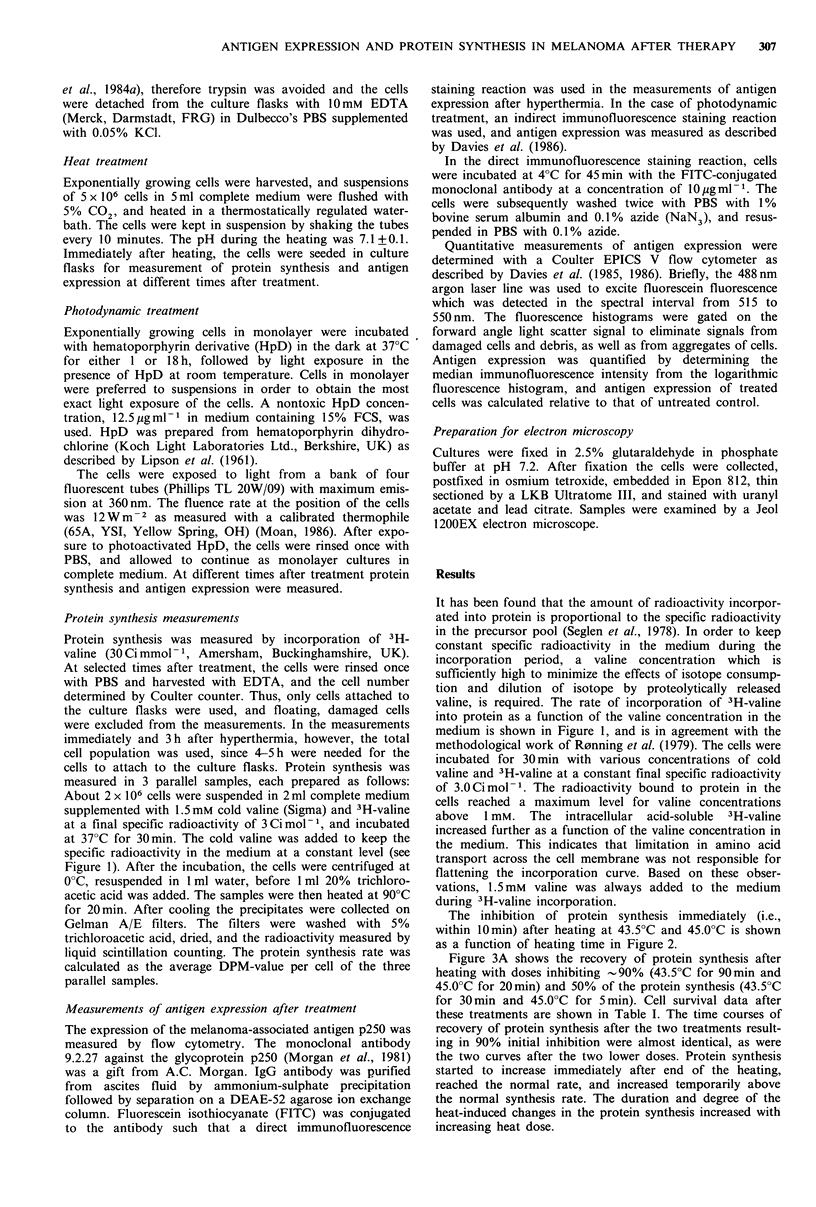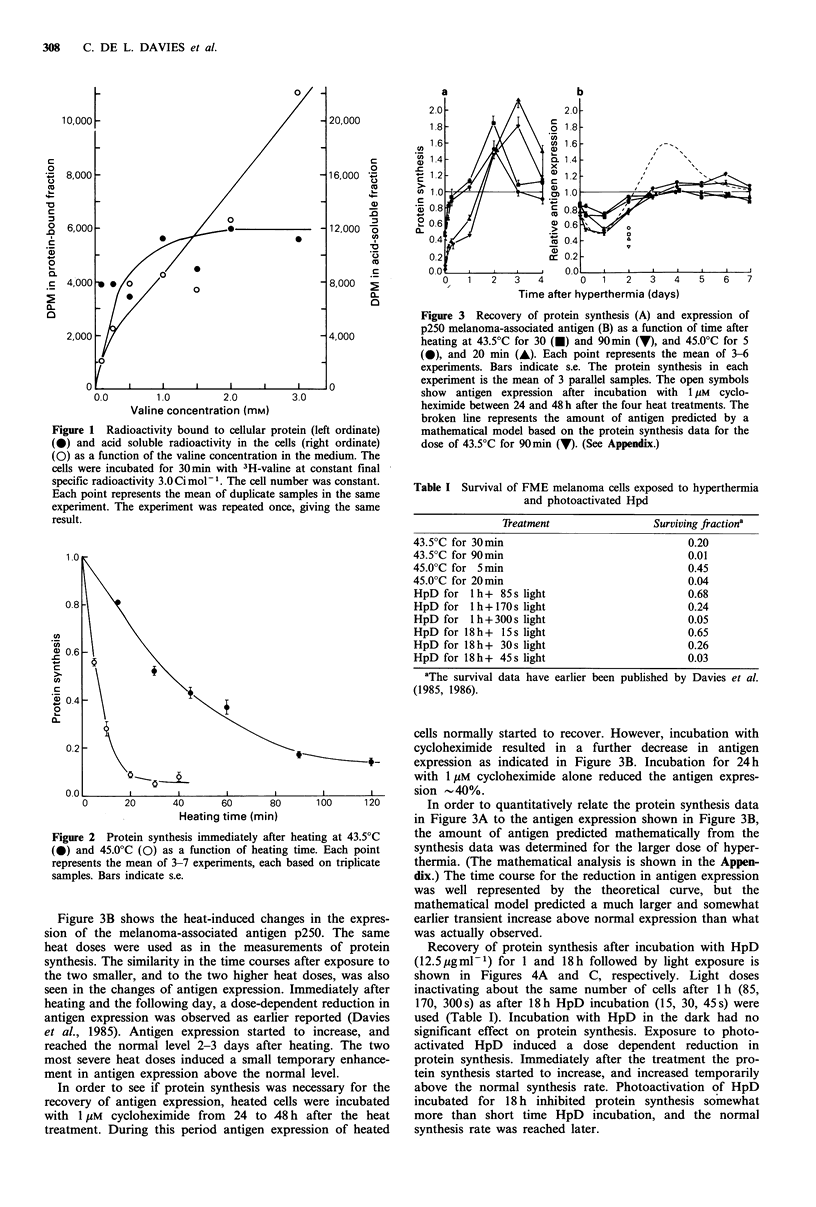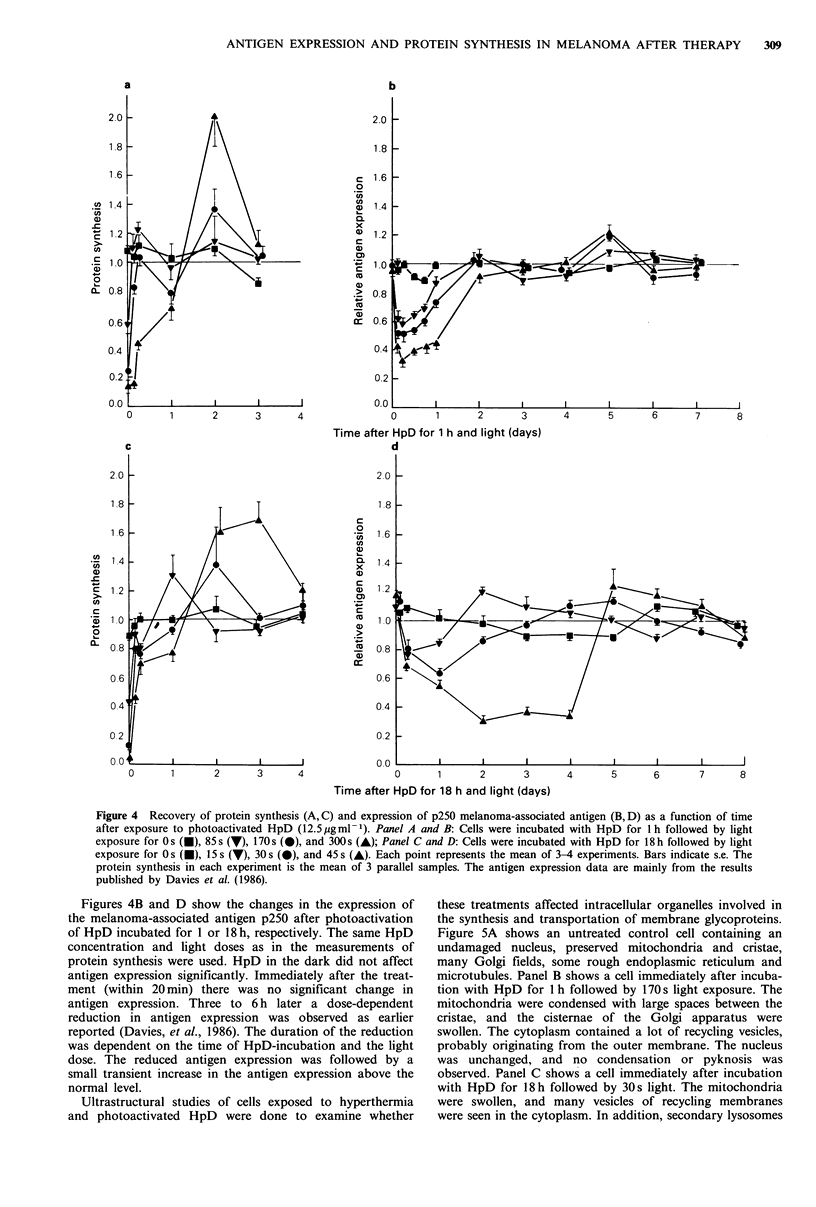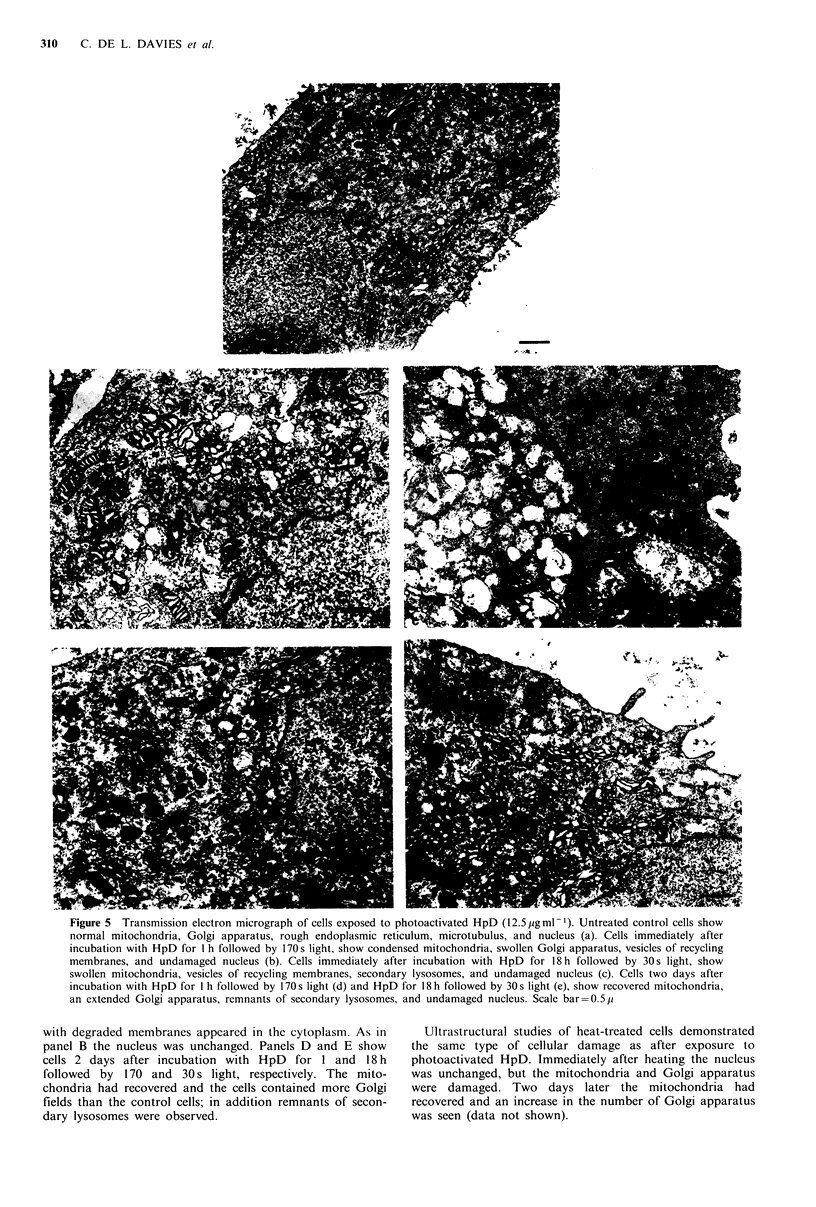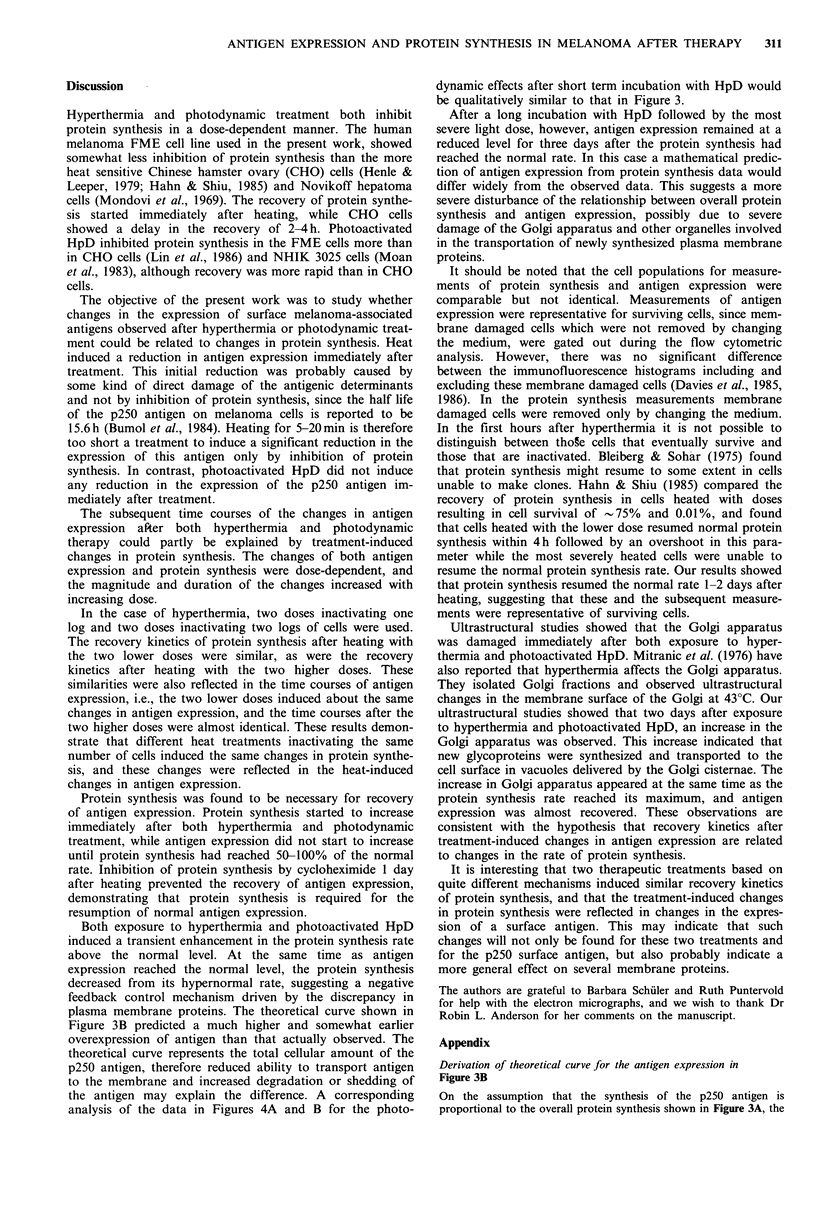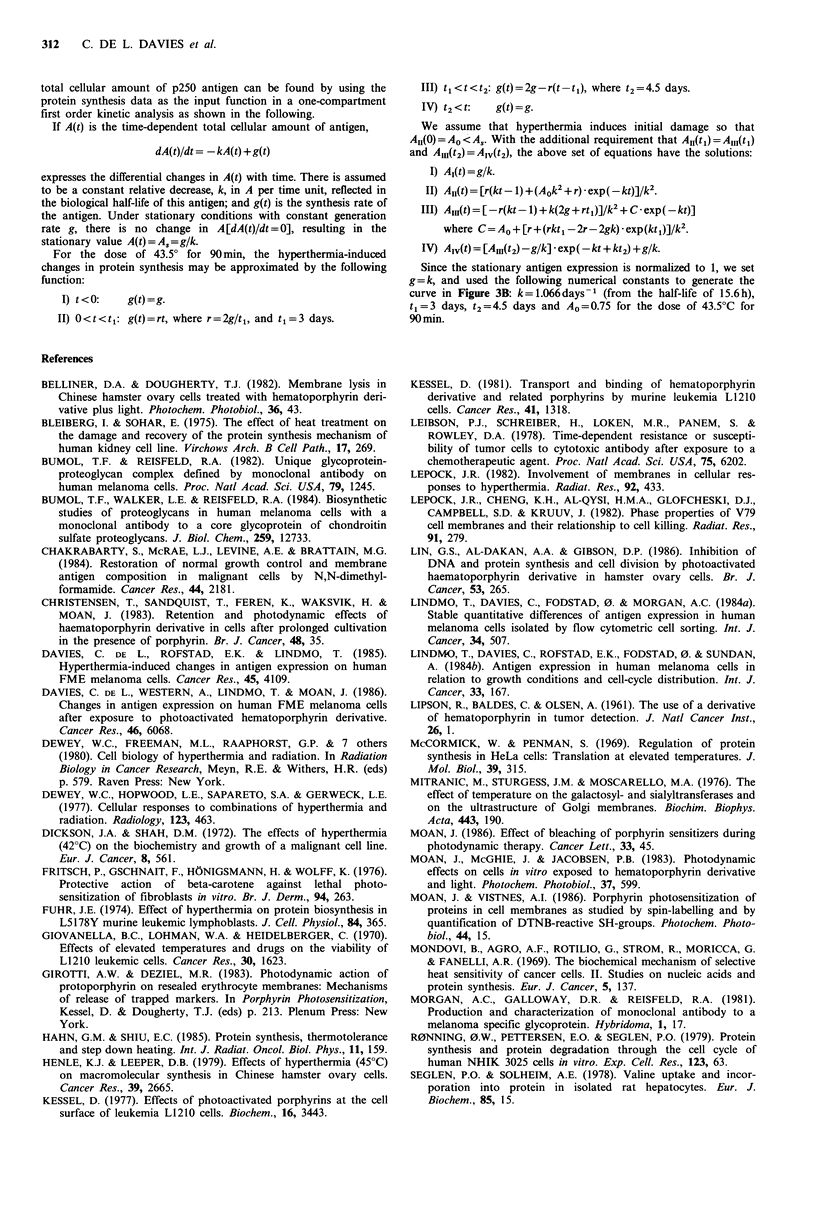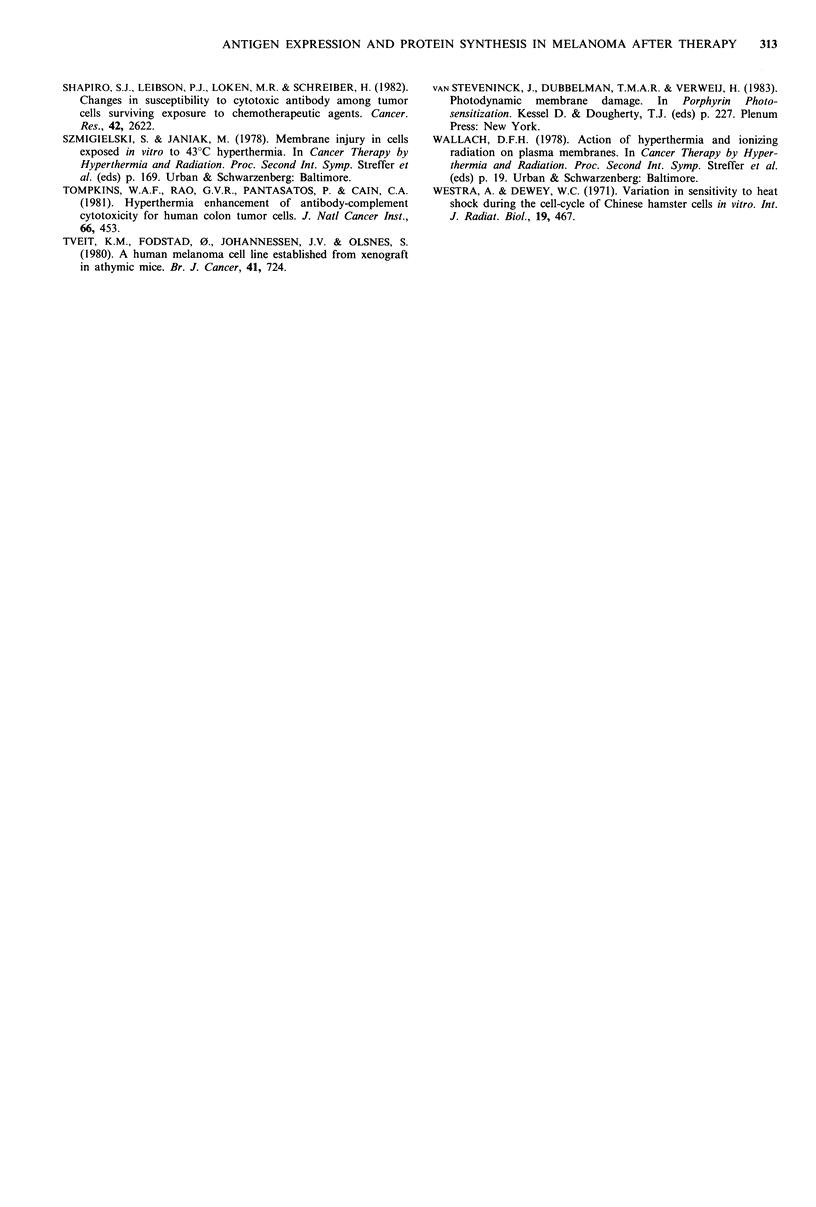# Relationship between changes in antigen expression and protein synthesis in human melanoma cells after hyperthermia and photodynamic treatment.

**DOI:** 10.1038/bjc.1988.209

**Published:** 1988-09

**Authors:** C. L. Davies, T. Ranheim, Z. Malik, E. K. Rofstad, J. Moan, T. Lindmo

**Affiliations:** Department of Biophysics, Institute for Cancer Research, Oslo, Norway.

## Abstract

**Images:**


*Br. J. Cancer* (1988) **58**, 306–313 © The Macmillan Press Ltd., 1988

### Relationship between changes in antigen expression and protein synthesis in human melanoma cells after hyperthermia and photodynamic treatment

C. de L. Davies[1], T. Ranheim[1], Z. Malik[2], E.K. Rofstad[1], J. Moan[1] & T. Lindmo[1]

[1]*Department of Biophysics, Institute for Cancer Research and the Norwegian Cancer Society, Montebello, 0310 Oslo 3, Norway; and* [2]*Life Sciences Department, Bar-Ilan University, Ramat-Gan 52100, Israel.*

**Summary** Hyperthermia and photoactivated hematoporphyrin derivative induce a dose-dependent reduction in the expression of the p250 surface melanoma-associated antigen on the human FME cell line. Expression of this glycoprotein antigen was quantitated by immunofluorescence flow cytometry based on the monoclonal antibody 9.2.27. Decrease in antigen expression was followed by a transient increase above the level for untreated cells, before normalization occurred about one week after treatment.

These treatment-induced changes in antigen expression could partly be explained by changes in protein synthesis. This conclusion was based on the following observations: Hyperthermia and photoactivated hematoporphyrin derivative both inhibited protein synthesis. The latter increased again rapidly to rates above normal until antigen expression reached normal level, whereupon the protein synthesis rate decreased to normal.

Inhibition of protein synthesis by cycloheximide 1 day after heating, prevented the recovery of antigen expression, demonstrating that protein synthesis is necessary for resumption of normal antigen expression.

The changes in both antigen expression and protein synthesis were dose-dependent, and the magnitude and duration of the changes increased with increasing dose. The time courses of the changes in protein synthesis after two different treatments which both inactivated two logs of cells were almost identical, as were the time courses after two lower heat doses inactivating one log of cells. These similarities were reflected in the changes in antigen expression.

At the same time as protein synthesis reached its maximum and antigen expression resumed normal level, an increase in the Golgi apparatus was observed ultrastructurally, indicating an increased synthesis rate and transportation of glycoproteins to the cell surface.

Hyperthermia induces a wide range of biochemical changes in mammalian cells, of which inhibition of protein synthesis is one of the most rapid and pronounced effects (McCormick & Penman, 1969; Mondovi *et al.*, 1969; Giovanella *et al.*, 1970; Dickson & Shah, 1972; Fuhr, 1974; Bleiberg & Sohar, 1975; Henle & Leeper, 1979). It has been suggested that the plasma membrane is a primary target for heat inactivation of cells (Szmigielski & Janiak, 1978; Wallach, 1978; Dewey *et al.*, 1980; Tompkins *et al.*, 1981), and that the irreversible changes in the membrane after hyperthermia are most likely due to protein damage (Westra & Dewey, 1971; Dewey *et al.*, 1977; Lepock, 1982; Lepock *et al.*, 1982).

Photodynamic therapy is another modality of cancer treatment where damage to the plasma membrane may lead to cell inactivation (Kessel, 1977; Bellnier & Dougherty, 1982), and membrane proteins are reported to be sensitive targets (Girotti & Deziel, 1983; van Steveninck *et al.*, 1983; Moan & Vistnes, 1986). The mechanisms for cell inactivation seem to some extent to depend on the photosensitizer concentration and on the incubation time with the photosensitizer (Fritsch *et al.*, 1976; Kessel, 1981; Christensen *et al.*, 1983). Photoactivation of porphyrin after incubation for a short time (1 h) has been reported to damage the membrane to a relatively greater degree than photoactivation of porphyrin after a long (22 h) incubation, using light doses inactivating the same number of cells (Christensen *et al.*, 1983; Moan *et al.*, 1983). A number of intracellular effects have been reported after photoactivation of porphyrins over short and long incubations. One of the most pronounced effects is inhibition of DNA synthesis. Inhibition of protein synthesis has also been observed, but this process is less sensitive (Moan *et al.*, 1983; Lin *et al.*, 1986).

Since plasma membrane proteins may be important targets in both hyperthermia and photodynamic therapy, we have studied changes in the expression of surface melanoma-associated antigens after these treatments (Davies *et al.*, 1985, 1986). Chemotherapy-induced changes in the expression of surface antigens on tumour cells have also been reported (Leibson *et al.*, 1978; Shapiro *et al.*, 1982; Chakrabarty *et al.*, 1984). A lot of work is going on to develop and characterize new monoclonal antibodies to tumour-associated antigens; and monoclonal antibodies including the one used in the present work, are being evaluated in clinical trials. In a clinical situation, therapy or disease monitoring based on monoclonal antibodies might be used in combination with various forms of cancer therapy. Therefore, it is of importance to know how the expression of tumour-associated antigens is affected by various treatments, e.g., for optimal time scheduling of antibody administration in relation to other treatments. The objective of the present work was to clarify the mechanisms behind treatment-induced changes in antigen expression, and investigate to what extent changes in protein synthesis could explain changes in antigen expression.

### Materials and methods

### *Cells*

The human melanoma cell line FME was established at our institute from a xenograft growing in athymic mice (Tveit *et al.*, 1980). Monolayer cultures of FME cells were grown at 37°C and 5% $CO_2$ in RPMI 1640 medium supplemented with 10% foetal calf serum (FCS), 1mM l-glutamine, and penicillin-streptomycin solution to a final concentration of 100 units $ml^{-1}$, all from Gibco (Paisley, UK). Grown under these conditions the doubling time was 18 h and the plating efficiency $0.60 \pm 0.15$. The medium was changed every second day, and always the day before treatment since medium starvation was found to cause a reduction in the antigen expression of these cells (Lindmo *et al.*, 1984*b*). The melanoma-associated antigen p250 (Bumol & Reisfeldt, 1982; Bumol *et al.*, 1984) is sensitive to trypsin treatment (Lindmo

Correspondence: C. de L. Davies.
Received 23 February 1988; and in revised form, 19 May 1988.

*et al.*, 1984*a*), therefore trypsin was avoided and the cells were detached from the culture flasks with 10 mM EDTA (Merck, Darmstadt, FRG) in Dulbecco's PBS supplemented with 0.05% KCl.

*Heat treatment*

Exponentially growing cells were harvested, and suspensions of $5 \times 10^6$ cells in 5 ml complete medium were flushed with 5% $CO_2$, and heated in a thermostatically regulated water-bath. The cells were kept in suspension by shaking the tubes every 10 minutes. The pH during the heating was $7.1 \pm 0.1$. Immediately after heating, the cells were seeded in culture flasks for measurement of protein synthesis and antigen expression at different times after treatment.

*Photodynamic treatment*

Exponentially growing cells in monolayer were incubated with hematoporphyrin derivative (HpD) in the dark at 37°C for either 1 or 18 h, followed by light exposure in the presence of HpD at room temperature. Cells in monolayer were preferred to suspensions in order to obtain the most exact light exposure of the cells. A nontoxic HpD concentration, 12.5 $\mu g\, ml^{-1}$ in medium containing 15% FCS, was used. HpD was prepared from hematoporphyrin dihydrochlorine (Koch Light Laboratories Ltd., Berkshire, UK) as described by Lipson *et al.* (1961).

The cells were exposed to light from a bank of four fluorescent tubes (Phillips TL 20W/09) with maximum emission at 360 nm. The fluence rate at the position of the cells was 12 $W\, m^{-2}$ as measured with a calibrated thermophile (65A, YSI, Yellow Spring, OH) (Moan, 1986). After exposure to photoactivated HpD, the cells were rinsed once with PBS, and allowed to continue as monolayer cultures in complete medium. At different times after treatment protein synthesis and antigen expression were measured.

*Protein synthesis measurements*

Protein synthesis was measured by incorporation of $^3$H-valine (30 $Ci\, mmol^{-1}$, Amersham, Buckinghamshire, UK). At selected times after treatment, the cells were rinsed once with PBS and harvested with EDTA, and the cell number determined by Coulter counter. Thus, only cells attached to the culture flasks were used, and floating, damaged cells were excluded from the measurements. In the measurements immediately and 3 h after hyperthermia, however, the total cell population was used, since 4–5 h were needed for the cells to attach to the culture flasks. Protein synthesis was measured in 3 parallel samples, each prepared as follows: About $2 \times 10^6$ cells were suspended in 2 ml complete medium supplemented with 1.5 mM cold valine (Sigma) and $^3$H-valine at a final specific radioactivity of 3 $Ci\, mol^{-1}$, and incubated at 37°C for 30 min. The cold valine was added to keep the specific radioactivity in the medium at a constant level (see Figure 1). After the incubation, the cells were centrifuged at 0°C, resuspended in 1 ml water, before 1 ml 20% trichloroacetic acid was added. The samples were then heated at 90°C for 20 min. After cooling the precipitates were collected on Gelman A/E filters. The filters were washed with 5% trichloroacetic acid, dried, and the radioactivity measured by liquid scintillation counting. The protein synthesis rate was calculated as the average DPM-value per cell of the three parallel samples.

*Measurements of antigen expression after treatment*

The expression of the melanoma-associated antigen p250 was measured by flow cytometry. The monoclonal antibody 9.2.27 against the glycoprotein p250 (Morgan *et al.*, 1981) was a gift from A.C. Morgan. IgG antibody was purified from ascites fluid by ammonium-sulphate precipitation followed by separation on a DEAE-52 agarose ion exchange column. Fluorescein isothiocyanate (FITC) was conjugated to the antibody such that a direct immunofluorescence staining reaction was used in the measurements of antigen expression after hyperthermia. In the case of photodynamic treatment, an indirect immunofluorescence staining reaction was used, and antigen expression was measured as described by Davies *et al.* (1986).

In the direct immunofluorescence staining reaction, cells were incubated at 4°C for 45 min with the FITC-conjugated monoclonal antibody at a concentration of 10 $\mu g\, ml^{-1}$. The cells were subsequently washed twice with PBS with 1% bovine serum albumin and 0.1% azide ($NaN_3$), and resuspended in PBS with 0.1% azide.

Quantitative measurements of antigen expression were determined with a Coulter EPICS V flow cytometer as described by Davies *et al.* (1985, 1986). Briefly, the 488 nm argon laser line was used to excite fluorescein fluorescence which was detected in the spectral interval from 515 to 550 nm. The fluorescence histograms were gated on the forward angle light scatter signal to eliminate signals from damaged cells and debris, as well as from aggregates of cells. Antigen expression was quantified by determining the median immunofluorescence intensity from the logarithmic fluorescence histogram, and antigen expression of treated cells was calculated relative to that of untreated control.

*Preparation for electron microscopy*

Cultures were fixed in 2.5% glutaraldehyde in phosphate buffer at pH 7.2. After fixation the cells were collected, postfixed in osmium tetroxide, embedded in Epon 812, thin sectioned by a LKB Ultratome III, and stained with uranyl acetate and lead citrate. Samples were examined by a Jeol 1200EX electron microscope.

### Results

It has been found that the amount of radioactivity incorporated into protein is proportional to the specific radioactivity in the precursor pool (Seglen *et al.*, 1978). In order to keep constant specific radioactivity in the medium during the incorporation period, a valine concentration which is sufficiently high to minimize the effects of isotope consumption and dilution of isotope by proteolytically released valine, is required. The rate of incorporation of $^3$H-valine into protein as a function of the valine concentration in the medium is shown in Figure 1, and is in agreement with the methodological work of Rønning *et al.* (1979). The cells were incubated for 30 min with various concentrations of cold valine and $^3$H-valine at a constant final specific radioactivity of 3.0 $Ci\, mol^{-1}$. The radioactivity bound to protein in the cells reached a maximum level for valine concentrations above 1 mM. The intracellular acid-soluble $^3$H-valine increased further as a function of the valine concentration in the medium. This indicates that limitation in amino acid transport across the cell membrane was not responsible for flattening the incorporation curve. Based on these observations, 1.5 mM valine was always added to the medium during $^3$H-valine incorporation.

The inhibition of protein synthesis immediately (i.e., within 10 min) after heating at 43.5°C and 45.0°C is shown as a function of heating time in Figure 2.

Figure 3A shows the recovery of protein synthesis after heating with doses inhibiting ~90% (43.5°C for 90 min and 45.0°C for 20 min) and 50% of the protein synthesis (43.5°C for 30 min and 45.0°C for 5 min). Cell survival data after these treatments are shown in Table I. The time courses of recovery of protein synthesis after the two treatments resulting in 90% initial inhibition were almost identical, as were the two curves after the two lower doses. Protein synthesis started to increase immediately after end of the heating, reached the normal rate, and increased temporarily above the normal synthesis rate. The duration and degree of the heat-induced changes in the protein synthesis increased with increasing heat dose.

**Figure 1** Radioactivity bound to cellular protein (left ordinate) (●) and acid soluble radioactivity in the cells (right ordinate) (○) as a function of the valine concentration in the medium. The cells were incubated for 30 min with $^3$H-valine at constant final specific radioactivity 3.0 Ci mol$^{-1}$. The cell number was constant. Each point represents the mean of duplicate samples in the same experiment. The experiment was repeated once, giving the same result.

**Figure 2** Protein synthesis immediately after heating at 43.5°C (●) and 45.0°C (○) as a function of heating time. Each point represents the mean of 3–7 experiments, each based on triplicate samples. Bars indicate s.e.

Figure 3B shows the heat-induced changes in the expression of the melanoma-associated antigen p250. The same heat doses were used as in the measurements of protein synthesis. The similarity in the time courses after exposure to the two smaller, and to the two higher heat doses, was also seen in the changes of antigen expression. Immediately after heating and the following day, a dose-dependent reduction in antigen expression was observed as earlier reported (Davies *et al.*, 1985). Antigen expression started to increase, and reached the normal level 2–3 days after heating. The two most severe heat doses induced a small temporary enhancement in antigen expression above the normal level.

In order to see if protein synthesis was necessary for the recovery of antigen expression, heated cells were incubated with 1 μM cycloheximide from 24 to 48 h after the heat treatment. During this period antigen expression of heated

**Figure 3** Recovery of protein synthesis (A) and expression of p250 melanoma-associated antigen (B) as a function of time after heating at 43.5°C for 30 (■) and 90 min (▼), and 45.0°C for 5 (●), and 20 min (▲). Each point represents the mean of 3–6 experiments. Bars indicate s.e. The protein synthesis in each experiment is the mean of 3 parallel samples. The open symbols show antigen expression after incubation with 1 μM cycloheximide between 24 and 48 h after the four heat treatments. The broken line represents the amount of antigen predicted by a mathematical model based on the protein synthesis data for the dose of 43.5°C for 90 min (▼). (See **Appendix**.)

**Table I** Survival of FME melanoma cells exposed to hyperthermia and photoactivated Hpd

| *Treatment* | *Surviving fraction*[a] |
|---|---|
| 43.5°C for 30 min | 0.20 |
| 43.5°C for 90 min | 0.01 |
| 45.0°C for 5 min | 0.45 |
| 45.0°C for 20 min | 0.04 |
| HpD for 1 h+ 85 s light | 0.68 |
| HpD for 1 h+170 s light | 0.24 |
| HpD for 1 h+300 s light | 0.05 |
| HpD for 18 h+ 15 s light | 0.65 |
| HpD for 18 h+ 30 s light | 0.26 |
| HpD for 18 h+ 45 s light | 0.03 |

[a]The survival data have earlier been published by Davies *et al.* (1985, 1986).

cells normally started to recover. However, incubation with cycloheximide resulted in a further decrease in antigen expression as indicated in Figure 3B. Incubation for 24 h with 1 μM cycloheximide alone reduced the antigen expression ~40%.

In order to quantitatively relate the protein synthesis data in Figure 3A to the antigen expression shown in Figure 3B, the amount of antigen predicted mathematically from the synthesis data was determined for the larger dose of hyperthermia. (The mathematical analysis is shown in the **Appendix**.) The time course for the reduction in antigen expression was well represented by the theoretical curve, but the mathematical model predicted a much larger and somewhat earlier transient increase above normal expression than what was actually observed.

Recovery of protein synthesis after incubation with HpD (12.5 μg ml$^{-1}$) for 1 and 18 h followed by light exposure is shown in Figures 4A and C, respectively. Light doses inactivating about the same number of cells after 1 h (85, 170, 300 s) as after 18 h HpD incubation (15, 30, 45 s) were used (Table I). Incubation with HpD in the dark had no significant effect on protein synthesis. Exposure to photoactivated HpD induced a dose dependent reduction in protein synthesis. Immediately after the treatment the protein synthesis started to increase, and increased temporarily above the normal synthesis rate. Photoactivation of HpD incubated for 18 h inhibited protein synthesis somewhat more than short time HpD incubation, and the normal synthesis rate was reached later.

**Figure 4** Recovery of protein synthesis (A, C) and expression of p250 melanoma-associated antigen (B, D) as a function of time after exposure to photoactivated HpD (12.5 $\mu g ml^{-1}$). *Panel A and B*: Cells were incubated with HpD for 1 h followed by light exposure for 0 s (■), 85 s (▼), 170 s (●), and 300 s (▲); *Panel C and D*: Cells were incubated with HpD for 18 h followed by light exposure for 0 s (■), 15 s (▼), 30 s (●), and 45 s (▲). Each point represents the mean of 3–4 experiments. Bars indicate s.e. The protein synthesis in each experiment is the mean of 3 parallel samples. The antigen expression data are mainly from the results published by Davies *et al.* (1986).

Figures 4B and D show the changes in the expression of the melanoma-associated antigen p250 after photoactivation of HpD incubated for 1 or 18 h, respectively. The same HpD concentration and light doses as in the measurements of protein synthesis were used. HpD in the dark did not affect antigen expression significantly. Immediately after the treatment (within 20 min) there was no significant change in antigen expression. Three to 6 h later a dose-dependent reduction in antigen expression was observed as earlier reported (Davies, *et al.*, 1986). The duration of the reduction was dependent on the time of HpD-incubation and the light dose. The reduced antigen expression was followed by a small transient increase in the antigen expression above the normal level.

Ultrastructural studies of cells exposed to hyperthermia and photoactivated HpD were done to examine whether these treatments affected intracellular organelles involved in the synthesis and transportation of membrane glycoproteins. Figure 5A shows an untreated control cell containing an undamaged nucleus, preserved mitochondria and cristae, many Golgi fields, some rough endoplasmic reticulum and microtubules. Panel B shows a cell immediately after incubation with HpD for 1 h followed by 170 s light exposure. The mitochondria were condensed with large spaces between the cristae, and the cisternae of the Golgi apparatus were swollen. The cytoplasm contained a lot of recycling vesicles, probably originating from the outer membrane. The nucleus was unchanged, and no condensation or pyknosis was observed. Panel C shows a cell immediately after incubation with HpD for 18 h followed by 30 s light. The mitochondria were swollen, and many vesicles of recycling membranes were seen in the cytoplasm. In addition, secondary lysosomes

**Figure 5** Transmission electron micrograph of cells exposed to photoactivated HpD ($12.5\,\mu g\,ml^{-1}$). Untreated control cells show normal mitochondria, Golgi apparatus, rough endoplasmic reticulum, microtubulus, and nucleus (a). Cells immediately after incubation with HpD for 1 h followed by 170 s light, show condensed mitochondria, swollen Golgi apparatus, vesicles of recycling membranes, and undamaged nucleus (b). Cells immediately after incubation with HpD for 18 h followed by 30 s light, show swollen mitochondria, vesicles of recycling membranes, secondary lysosomes, and undamaged nucleus (c). Cells two days after incubation with HpD for 1 h followed by 170 s light (d) and HpD for 18 h followed by 30 s light (e), show recovered mitochondria, an extended Golgi apparatus, remnants of secondary lysosomes, and undamaged nucleus. Scale bar = $0.5\,\mu$

with degraded membranes appeared in the cytoplasm. As in panel B the nucleus was unchanged. Panels D and E show cells 2 days after incubation with HpD for 1 and 18 h followed by 170 and 30 s light, respectively. The mitochondria had recovered and the cells contained more Golgi fields than the control cells; in addition remnants of secondary lysosomes were observed.

Ultrastructural studies of heat-treated cells demonstrated the same type of cellular damage as after exposure to photoactivated HpD. Immediately after heating the nucleus was unchanged, but the mitochondria and Golgi apparatus were damaged. Two days later the mitochondria had recovered and an increase in the number of Golgi apparatus was seen (data not shown).

### Discussion

Hyperthermia and photodynamic treatment both inhibit protein synthesis in a dose-dependent manner. The human melanoma FME cell line used in the present work, showed somewhat less inhibition of protein synthesis than the more heat sensitive Chinese hamster ovary (CHO) cells (Henle & Leeper, 1979; Hahn & Shiu, 1985) and Novikoff hepatoma cells (Mondovi *et al*., 1969). The recovery of protein synthesis started immediately after heating, while CHO cells showed a delay in the recovery of 2–4 h. Photoactivated HpD inhibited protein synthesis in the FME cells more than in CHO cells (Lin *et al*., 1986) and NHIK 3025 cells (Moan *et al*., 1983), although recovery was more rapid than in CHO cells.

The objective of the present work was to study whether changes in the expression of surface melanoma-associated antigens observed after hyperthermia or photodynamic treatment could be related to changes in protein synthesis. Heat induced a reduction in antigen expression immediately after treatment. This initial reduction was probably caused by some kind of direct damage of the antigenic determinants and not by inhibition of protein synthesis, since the half life of the p250 antigen on melanoma cells is reported to be 15.6 h (Bumol *et al*., 1984). Heating for 5–20 min is therefore too short a treatment to induce a significant reduction in the expression of this antigen only by inhibition of protein synthesis. In contrast, photoactivated HpD did not induce any reduction in the expression of the p250 antigen immediately after treatment.

The subsequent time courses of the changes in antigen expression after both hyperthermia and photodynamic therapy could partly be explained by treatment-induced changes in protein synthesis. The changes of both antigen expression and protein synthesis were dose-dependent, and the magnitude and duration of the changes increased with increasing dose.

In the case of hyperthermia, two doses inactivating one log and two doses inactivating two logs of cells were used. The recovery kinetics of protein synthesis after heating with the two lower doses were similar, as were the recovery kinetics after heating with the two higher doses. These similarities were also reflected in the time courses of antigen expression, i.e., the two lower doses induced about the same changes in antigen expression, and the time courses after the two higher doses were almost identical. These results demonstrate that different heat treatments inactivating the same number of cells induced the same changes in protein synthesis, and these changes were reflected in the heat-induced changes in antigen expression.

Protein synthesis was found to be necessary for recovery of antigen expression. Protein synthesis started to increase immediately after both hyperthermia and photodynamic treatment, while antigen expression did not start to increase until protein synthesis had reached 50–100% of the normal rate. Inhibition of protein synthesis by cycloheximide 1 day after heating prevented the recovery of antigen expression, demonstrating that protein synthesis is required for the resumption of normal antigen expression.

Both exposure to hyperthermia and photoactivated HpD induced a transient enhancement in the protein synthesis rate above the normal level. At the same time as antigen expression reached the normal level, the protein synthesis decreased from its hypernormal rate, suggesting a negative feedback control mechanism driven by the discrepancy in plasma membrane proteins. The theoretical curve shown in Figure 3B predicted a much higher and somewhat earlier overexpression of antigen than that actually observed. The theoretical curve represents the total cellular amount of the p250 antigen, therefore reduced ability to transport antigen to the membrane and increased degradation or shedding of the antigen may explain the difference. A corresponding analysis of the data in Figures 4A and B for the photodynamic effects after short term incubation with HpD would be qualitatively similar to that in Figure 3.

After a long incubation with HpD followed by the most severe light dose, however, antigen expression remained at a reduced level for three days after the protein synthesis had reached the normal rate. In this case a mathematical prediction of antigen expression from protein synthesis data would differ widely from the observed data. This suggests a more severe disturbance of the relationship between overall protein synthesis and antigen expression, possibly due to severe damage of the Golgi apparatus and other organelles involved in the transportation of newly synthesized plasma membrane proteins.

It should be noted that the cell populations for measurements of protein synthesis and antigen expression were comparable but not identical. Measurements of antigen expression were representative for surviving cells, since membrane damaged cells which were not removed by changing the medium, were gated out during the flow cytometric analysis. However, there was no significant difference between the immunofluorescence histograms including and excluding these membrane damaged cells (Davies *et al*., 1985, 1986). In the protein synthesis measurements membrane damaged cells were removed only by changing the medium. In the first hours after hyperthermia it is not possible to distinguish between those cells that eventually survive and those that are inactivated. Bleiberg & Sohar (1975) found that protein synthesis might resume to some extent in cells unable to make clones. Hahn & Shiu (1985) compared the recovery of protein synthesis in cells heated with doses resulting in cell survival of ~75% and 0.01%, and found that cells heated with the lower dose resumed normal protein synthesis within 4 h followed by an overshoot in this parameter while the most severely heated cells were unable to resume the normal protein synthesis rate. Our results showed that protein synthesis resumed the normal rate 1–2 days after heating, suggesting that these and the subsequent measurements were representative of surviving cells.

Ultrastructural studies showed that the Golgi apparatus was damaged immediately after both exposure to hyperthermia and photoactivated HpD. Mitranic *et al*. (1976) have also reported that hyperthermia affects the Golgi apparatus. They isolated Golgi fractions and observed ultrastructural changes in the membrane surface of the Golgi at 43°C. Our ultrastructural studies showed that two days after exposure to hyperthermia and photoactivated HpD, an increase in the Golgi apparatus was observed. This increase indicated that new glycoproteins were synthesized and transported to the cell surface in vacuoles delivered by the Golgi cisternae. The increase in Golgi apparatus appeared at the same time as the protein synthesis rate reached its maximum, and antigen expression was almost recovered. These observations are consistent with the hypothesis that recovery kinetics after treatment-induced changes in antigen expression are related to changes in the rate of protein synthesis.

It is interesting that two therapeutic treatments based on quite different mechanisms induced similar recovery kinetics of protein synthesis, and that the treatment-induced changes in protein synthesis were reflected in changes in the expression of a surface antigen. This may indicate that such changes will not only be found for these two treatments and for the p250 surface antigen, but also probably indicate a more general effect on several membrane proteins.

The authors are grateful to Barbara Schüler and Ruth Puntervold for help with the electron micrographs, and we wish to thank Dr Robin L. Anderson for her comments on the manuscript.

### Appendix

*Derivation of theoretical curve for the antigen expression in* **Figure 3B**

On the assumption that the synthesis of the p250 antigen is proportional to the overall protein synthesis shown in **Figure 3A**, the total cellular amount of p250 antigen can be found by using the protein synthesis data as the input function in a one-compartment first order kinetic analysis as shown in the following.

If $A(t)$ is the time-dependent total cellular amount of antigen,

$$dA(t)/dt = -kA(t) + g(t)$$

expresses the differential changes in $A(t)$ with time. There is assumed to be a constant relative decrease, $k$, in $A$ per time unit, reflected in the biological half-life of this antigen; and $g(t)$ is the synthesis rate of the antigen. Under stationary conditions with constant generation rate $g$, there is no change in $A[dA(t)/dt = 0]$, resulting in the stationary value $A(t) = A_s = g/k$.

For the dose of 43.5° for 90 min, the hyperthermia-induced changes in protein synthesis may be approximated by the following function:

I) $t < 0$: $g(t) = g$.

II) $0 < t < t_1$: $g(t) = rt$, where $r = 2g/t_1$, and $t_1 = 3$ days.

III) $t_1 < t < t_2$: $g(t) = 2g - r(t - t_1)$, where $t_2 = 4.5$ days.

IV) $t_2 < t$: $g(t) = g$.

We assume that hyperthermia induces initial damage so that $A_{II}(0) = A_0 < A_s$. With the additional requirement that $A_{II}(t_1) = A_{III}(t_1)$ and $A_{III}(t_2) = A_{IV}(t_2)$, the above set of equations have the solutions:

I) $A_I(t) = g/k$.

II) $A_{II}(t) = [r(kt-1) + (A_0k^2 + r)\cdot \exp(-kt)]/k^2$.

III) $A_{III}(t) = [-r(kt-1) + k(2g + rt_1)]/k^2 + C\cdot \exp(-kt)]$

where $C = A_0 + [r + (rkt_1 - 2r - 2gk)\cdot \exp(kt_1)]/k^2$.

IV) $A_{IV}(t) = [A_{III}(t_2) - g/k]\cdot \exp(-kt + kt_2) + g/k$.

Since the stationary antigen expression is normalized to 1, we set $g = k$, and used the following numerical constants to generate the curve in **Figure 3B**: $k = 1.066\,\text{days}^{-1}$ (from the half-life of 15.6 h), $t_1 = 3$ days, $t_2 = 4.5$ days and $A_0 = 0.75$ for the dose of 43.5°C for 90 min.

### References

BELLINER, D.A. & DOUGHERTY, T.J. (1982). Membrane lysis in Chinese hamster ovary cells treated with hematoporphyrin derivative plus light. *Photochem. Photobiol.*, **36**, 43.

BLEIBERG, I. & SOHAR, E. (1975). The effect of heat treatment on the damage and recovery of the protein synthesis mechanism of human kidney cell line. *Virchows Arch. B Cell Path.*, **17**, 269.

BUMOL, T.F. & REISFELD, R.A. (1982). Unique glycoprotein-proteoglycan complex defined by monoclonal antibody on human melanoma cells. *Proc. Natl Acad. Sci. USA*, **79**, 1245.

BUMOL, T.F., WALKER, L.E. & REISFELD, R.A. (1984). Biosynthetic studies of proteoglycans in human melanoma cells with a monoclonal antibody to a core glycoprotein of chondroitin sulfate proteoglycans. *J. Biol. Chem.*, **259**, 12733.

CHAKRABARTY, S., MCRAE, L.J., LEVINE, A.E. & BRATTAIN, M.G. (1984). Restoration of normal growth control and membrane antigen composition in malignant cells by N,N-dimethylformamide. *Cancer Res.*, **44**, 2181.

CHRISTENSEN, T., SANDQUIST, T., FEREN, K., WAKSVIK, H. & MOAN, J. (1983). Retention and photodynamic effects of haematoporphyrin derivative in cells after prolonged cultivation in the presence of porphyrin. *Br. J. Cancer*, **48**, 35.

DAVIES, C. DE L., ROFSTAD, E.K. & LINDMO, T. (1985). Hyperthermia-induced changes in antigen expression on human FME melanoma cells. *Cancer Res.*, **45**, 4109.

DAVIES, C. DE L., WESTERN, A., LINDMO, T. & MOAN, J. (1986). Changes in antigen expression on human FME melanoma cells after exposure to photoactivated hematoporphyrin derivative. *Cancer Res.*, **46**, 6068.

DEWEY, W.C., FREEMAN, M.L., RAAPHORST, G.P. & 7 others (1980). Cell biology of hyperthermia and radiation. In *Radiation Biology in Cancer Research*, Meyn, R.E. & Withers, H.R. (eds) p. 579. Raven Press: New York.

DEWEY, W.C., HOPWOOD, L.E., SAPARETO, S.A. & GERWECK, L.E. (1977). Cellular responses to combinations of hyperthermia and radiation. *Radiology*, **123**, 463.

DICKSON, J.A. & SHAH, D.M. (1972). The effects of hyperthermia (42°C) on the biochemistry and growth of a malignant cell line. *Eur. J. Cancer*, **8**, 561.

FRITSCH, P., GSCHNAIT, F., HÖNIGSMANN, H. & WOLFF, K. (1976). Protective action of beta-carotene against lethal photosensitization of fibroblasts *in vitro*. *Br. J. Derm.*, **94**, 263.

FUHR, J.E. (1974). Effect of hyperthermia on protein biosynthesis in L5178Y murine leukemic lymphoblasts. *J. Cell. Physiol.*, **84**, 365.

GIOVANELLA, B.C., LOHMAN, W.A. & HEIDELBERGER, C. (1970). Effects of elevated temperatures and drugs on the viability of L1210 leukemic cells. *Cancer Res.*, **30**, 1623.

GIROTTI, A.W. & DEZIEL, M.R. (1983). Photodynamic action of protoporphyrin on resealed erythrocyte membranes: Mechanisms of release of trapped markers. In *Porphyrin Photosensitization*, Kessel, D. & Dougherty, T.J. (eds) p. 213. Plenum Press: New York.

HAHN, G.M. & SHIU, E.C. (1985). Protein synthesis, thermotolerance and step down heating. *Int. J. Radiat. Oncol. Biol. Phys.*, **11**, 159.

HENLE, K.J. & LEEPER, D.B. (1979). Effects of hyperthermia (45°C) on macromolecular synthesis in Chinese hamster ovary cells. *Cancer Res.*, **39**, 2665.

KESSEL, D. (1977). Effects of photoactivated porphyrins at the cell surface of leukemia L1210 cells. *Biochem.*, **16**, 3443.

KESSEL, D. (1981). Transport and binding of hematoporphyrin derivative and related porphyrins by murine leukemia L1210 cells. *Cancer Res.*, **41**, 1318.

LEIBSON, P.J., SCHREIBER, H., LOKEN, M.R., PANEM, S. & ROWLEY, D.A. (1978). Time-dependent resistance or susceptibility of tumor cells to cytotoxic antibody after exposure to a chemotherapeutic agent. *Proc. Natl Acad. Sci. USA*, **75**, 6202.

LEPOCK, J.R. (1982). Involvement of membranes in cellular responses to hyperthermia. *Radiat. Res.*, **92**, 433.

LEPOCK, J.R., CHENG, K.H., AL-QYSI, H.M.A., GLOFCHESKI, D.J., CAMPBELL, S.D. & KRUUV, J. (1982). Phase properties of V79 cell membranes and their relationship to cell killing. *Radiat. Res.*, **91**, 279.

LIN, G.S., AL-DAKAN, A.A. & GIBSON, D.P. (1986). Inhibition of DNA and protein synthesis and cell division by photoactivated haematoporphyrin derivative in hamster ovary cells. *Br. J. Cancer*, **53**, 265.

LINDMO, T., DAVIES, C., FODSTAD, Ø. & MORGAN, A.C. (1984*a*). Stable quantitative differences of antigen expression in human melanoma cells isolated by flow cytometric cell sorting. *Int. J. Cancer*, **34**, 507.

LINDMO, T., DAVIES, C., ROFSTAD, E.K., FODSTAD, Ø. & SUNDAN, A. (1984*b*). Antigen expression in human melanoma cells in relation to growth conditions and cell-cycle distribution. *Int. J. Cancer*, **33**, 167.

LIPSON, R., BALDES, C. & OLSEN, A. (1961). The use of a derivative of hematoporphyrin in tumor detection. *J. Natl Cancer Inst.*, **26**, 1.

MCCORMICK, W. & PENMAN, S. (1969). Regulation of protein synthesis in HeLa cells: Translation at elevated temperatures. *J. Mol. Biol.*, **39**, 315.

MITRANIC, M., STURGESS, J.M. & MOSCARELLO, M.A. (1976). The effect of temperature on the galactosyl- and sialyltransferases and on the ultrastructure of Golgi membranes. *Biochim. Biophys. Acta*, **443**, 190.

MOAN, J. (1986). Effect of bleaching of porphyrin sensitizers during photodynamic therapy. *Cancer Lett.*, **33**, 45.

MOAN, J., MCGHIE, J. & JACOBSEN, P.B. (1983). Photodynamic effects on cells *in vitro* exposed to hematoporphyrin derivative and light. *Photochem. Photobiol.*, **37**, 599.

MOAN, J. & VISTNES, A.I. (1986). Porphyrin photosensitization of proteins in cell membranes as studied by spin-labelling and by quantification of DTNB-reactive SH-groups. *Photochem. Photobiol.*, **44**, 15.

MONDOVI, B., AGRO, A.F., ROTILIO, G., STROM, R., MORICCA, G. & FANELLI, A.R. (1969). The biochemical mechanism of selective heat sensitivity of cancer cells. II. Studies on nucleic acids and protein synthesis. *Eur. J. Cancer*, **5**, 137.

MORGAN, A.C., GALLOWAY, D.R. & REISFELD, R.A. (1981). Production and characterization of monoclonal antibody to a melanoma specific glycoprotein. *Hybridoma*, **1**, 17.

RØNNING, Ø.W., PETTERSEN, E.O. & SEGLEN, P.O. (1979). Protein synthesis and protein degradation through the cell cycle of human NHIK 3025 cells *in vitro*. *Exp. Cell. Res.*, **123**, 63.

SEGLEN, P.O. & SOLHEIM, A.E. (1978). Valine uptake and incorporation into protein in isolated rat hepatocytes. *Eur. J. Biochem.*, **85**, 15.

SHAPIRO, S.J., LEIBSON, P.J., LOKEN, M.R. & SCHREIBER, H. (1982). Changes in susceptibility to cytotoxic antibody among tumor cells surviving exposure to chemotherapeutic agents. *Cancer. Res.*, **42,** 2622.

SZMIGIELSKI, S. & JANIAK, M. (1978). Membrane injury in cells exposed *in vitro* to 43°C hyperthermia. In *Cancer Therapy by Hyperthermia and Radiation. Proc. Second Int. Symp.* Streffer *et al.* (eds) p. 169. Urban & Schwarzenberg: Baltimore.

TOMPKINS, W.A.F., RAO, G.V.R., PANTASATOS, P. & CAIN, C.A. (1981). Hyperthermia enhancement of antibody-complement cytotoxicity for human colon tumor cells. *J. Natl Cancer Inst.*, **66,** 453.

TVEIT, K.M., FODSTAD, Ø., JOHANNESSEN, J.V. & OLSNES, S. (1980). A human melanoma cell line established from xenograft in athymic mice. *Br. J. Cancer*, **41,** 724.

VAN STEVENINCK, J., DUBBELMAN, T.M.A.R. & VERWEIJ, H. (1983). Photodynamic membrane damage. In *Porphyrin Photosensitization.* Kessel D. & Dougherty, T.J. (eds) p. 227. Plenum Press: New York.

WALLACH, D.F.H. (1978). Action of hyperthermia and ionizing radiation on plasma membranes. In *Cancer Therapy by Hyperthermia and Radiation. Proc. Second Int. Symp.* Streffer *et al.* (eds) p. 19. Urban & Schwarzenberg: Baltimore.

WESTRA, A. & DEWEY, W.C. (1971). Variation in sensitivity to heat shock during the cell-cycle of Chinese hamster cells *in vitro. Int. J. Radiat. Biol.*, **19,** 467.